# Temperature-Insensitive Structure Design of Micromachined Resonant Accelerometers

**DOI:** 10.3390/s19071544

**Published:** 2019-03-30

**Authors:** Yonggang Yin, Zhengxiang Fang, Yunfeng Liu, Fengtian Han

**Affiliations:** Department of Precision Instrument, Tsinghua University, Beijing 100084, China; yinyg14@mails.tsinghua.edu.cn (Y.Y.); fang-zx16@mails.tsinghua.edu.cn (Z.F.)

**Keywords:** microelectromechanical systems (MEMS), resonant accelerometer, silicon on glass (SOG), temperature sensitivity, thermal stress

## Abstract

Micromachined resonant accelerometers (MRAs), especially those devices fabricated by silicon on glass technology, suffer from temperature drift error caused by inherent thermal stress. This paper proposes two structure designs to attenuate the effect of thermal stress. The first MRA structure is realized by optimizing the locations of the bonding anchors and utilizing a special-shaped substrate to isolate the thermal stress generated during the die attach process. The second structure is designed using an isolation frame fixed by a single anchor to replace all dispersed anchors associated with the suspension beams and micro-levers. Simulated and experimental results show that both of the MRA structures can effectively reduce the thermal stress effect. The experimental results on one MRA prototype indicate that the differential temperature sensitivity reduces down to 1.9 μg/°C and its 15-day bias stability reaches 1.4 μg.

## 1. Introduction

With the rapid development of microelectromechanical systems (MEMS) technology over three decades, micromachined accelerometers have shown potential for high-precision applications, such as strategic-grade navigation [1], seismology [2,3,4,5] and gravimetry [6,7]. Based on the force-frequency characteristics of high-Q resonators, micromachined resonant accelerometer (MRA) is very attractive due to its high resolution and large dynamic range [8,9,10]. However, the temperature-dependent drifts of the bias and scale factor limit the long-term stability of those high-performance MRAs. There are two major sources of temperature drift dominated by the sensing structure [11]. Firstly, the Young’s modulus of silicon [12] varies with temperature change, which will directly result in the natural frequency drift of the resonator [13]. Secondly, the mismatch of the coefficients of thermal expansion (CTEs) between the silicon structure and its substrate will inevitably generate thermal stress.

Several methods have been reported in the literature to reduce the two errors mentioned above. For silicon micromechanical resonators used as frequency and timing devices, the temperature drift of Young’s modulus can be compensated effectively by doping the silicon with dopants [14,15] or using a composite temperature-insensitive structure, such as silicon dioxide and silicon, whose Young’s modulus changes with temperature in opposite directions [13,16]. Nevertheless, the methods above are not generally utilized in MRAs because the temperature drift of Young’s modulus can be greatly decreased by a differential structure design comprising two symmetric resonators [17,18].

Currently, the effect of the thermal stress becomes the main limitation on the MRA’s output stability. Michel [19] designed a silicon resonator on a glass substrate and proposed a method to counteract the effect of the thermal stress and the Young’s modulus by adjusting the distance between resonator anchors. Since the change in Young’s modulus will make the resonant frequency decrease as the temperature rises, the frequency drift induced by thermal stress should be positive by design. In a similar manner, Myers et al. [20] designed a silicon carbide resonator on a single-crystalline silicon substrate to counteract the two kinds of drifts. However, the die attach process during the device packaging will generate unpredictable thermal stress [21], which could deviate the theoretical result of the temperature insensitive point greatly. Zhao et al. [22] and Zhang et al. [23] proposed a frame structure to decrease the amount of anchors. In these MRAs, the resonators and the micro-levers are connected to the fixed frame instead of anchors so that the thermal stress from the substrate will not act on the resonators directly. However, the two resonators in this design still suffer from large thermal stress because the deformation of the frame is very sensitive to the temperature change. Yang et al. [24] proposed an isolation platform for inertial sensors, which can reduce the thermal stress from the package and printed circuit board. However, the adhesive layer over the platform still has a remarkable effect on the sensor structure. Shin et al. [25,26,27] demonstrated a differential resonant accelerometer with a single-point anchor. This design can greatly eliminate frequency fluctuations, due to package stress. Nevertheless, the structure may be fragile and suffers from large cross-axis coupling error as a small anchor was used to support the proof mass where the resonant beam acts as a cantilever.

Our preliminary results on the temperature-insensitive structure with thermal stress isolation were presented in Reference [28]. In this paper, more theoretical analysis and the latest experimental results are introduced and demonstrated the low temperature drift performance. Moreover, a novel structure is also proposed using an isolation frame fixed by a single anchor to replace all dispersed anchors associated with the suspension beams and micro-levers. These two structure designs aim to minimize the effect of thermal stress and improve the temperature drift consistency of the two resonators operating in a differential output.

## 2. Operation Principle of the MRA

**Figure 1:**
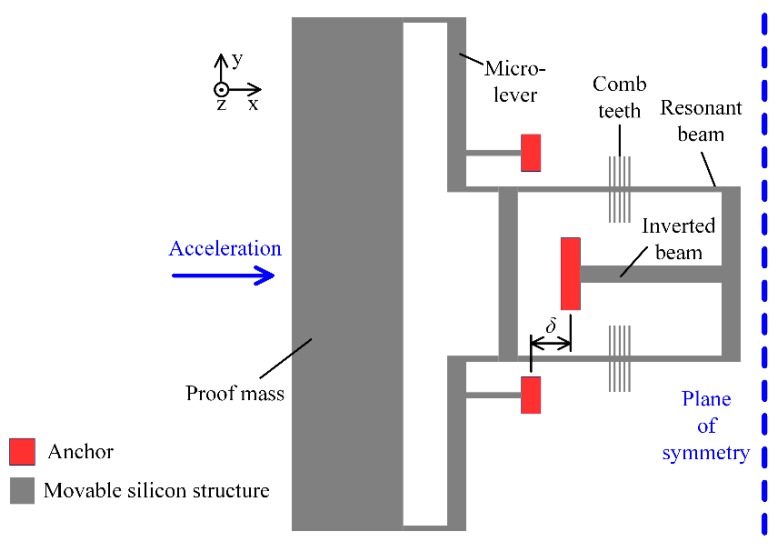
A schematic of the sensing structure (half of the symmetric device).

The structure of our MRA is fully symmetric and half of the schematic structure is illustrated in Figure 1. The movable silicon structure is bonded to a glass substrate via a set of anchors. The input acceleration along the sensitive axis (*x*-axis) will make the proof mass displace away from its nominal position. Then the inertial force is amplified by the micro-lever system in order to achieve high sensitivity. Finally, the double-ended tuning fork (DETF) resonator in Figure 1 is stretched, which will increase its natural frequency. On the other hand, the symmetric DETF is compressed and its natural frequency will be decreased. Within a certain measurement range, the differential frequency of the two resonators is proportional to the input acceleration. This differential output will cancel most of the common error sources of the two resonators. Note that the two resonant beams in each DETF vibrate in reversed-phase mode, which can improve the Q-factor of the resonators.

Consider the resonant beam as a fixed-fixed beam, its resonant frequency can be given by

(1) $$f_{0}=\frac{1}{2\pi }\sqrt{\frac{199EI}{ml^{3}}},$$

where *E* is the Young’s modulus of silicon, *I* is the inertia moment of the cross section, *l* is the length of the beam and *m* is the equivalent mass. Then the temperature coefficient of the resonant frequency caused by the Young’s modulus is

(2) $$C_{E}=\frac{df_{0}}{dT}=\frac{f_{0}}{2}\frac{dE}{EdT},$$

where *T* is the temperature. When an external force *P* is applied along the beam, the resonant frequency will be

(3) $$f_{P}=f_{0}\sqrt{1+\frac{Pl^{2}}{4\pi^{2}EI}}\approx f_{0}(1+\frac{Pl^{2}}{8\pi^{2}EI}).$$

The temperature coefficient of frequency change caused by the external force is

(4) $$C_{P}=\frac{df_{P}}{dT}=\frac{f_{0}l^{2}}{8\pi^{2}EI}\frac{dP}{dT}.$$

In this work, the MRA devices were fabricated based on silicon on glass (SOG) process [1,29]. Since the CTE of the glass substrate (*α*_g_) is slightly larger than that of the silicon structure (*α*_s_), the *x*-axis distance between the anchors (*δ*) will increase as the temperature rises. In this case, the tensile thermal force will be generated in the resonant beam, as modeled in

(5) $$P=k\delta (\alpha_{g}-\alpha_{s})T.$$

where *k* is the axial stiffness of the resonant beam.

Worse still, during the subsequent die attach process by packaging the glass substrate on a ceramic base, large thermal expansion of the adhesive layer will deteriorate the theoretical frequency drift results greatly. Its effect can be equivalent to increase the CTE of the glass substrate (*α*_g_) in (5). Additionally, it is difficult to maintain a uniform thickness of the adhesive layer, which will increase the differential frequency error, due to inconsistent temperature responses of the two resonators.

In order to reduce the effect of the thermal stress and improve the temperature drift consistency of the two differential resonators in the MRA device, two structure designs are proposed in this work.

## 3. Design A: Anchors’ Location Optimization and Isolation Substrate

**Figure 2:**
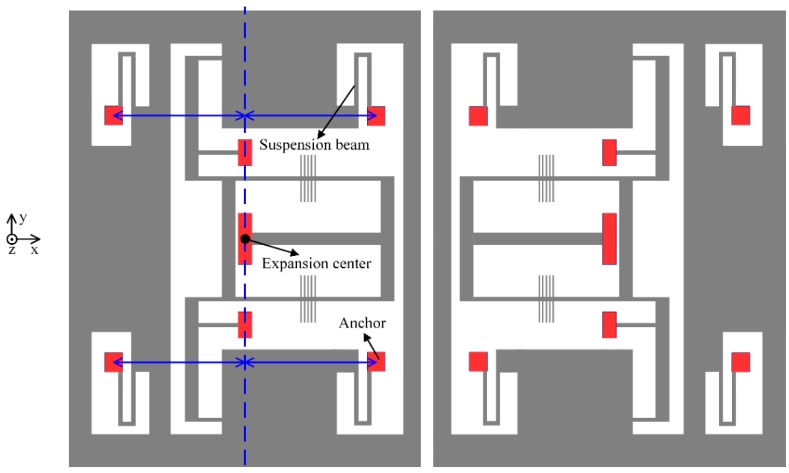
The schematic structure with optimized anchor locations.

In the first design, the locations of the bonding anchors are optimized to decrease the thermal stress between the glass substrate and the silicon structure. In principle, the thermal stress coefficient (*k*) is dominated by *δ*. If *δ* = 0, which can be implemented by using an inverted beam design (as shown in Figure 1), the thermal stress along the acceleration-sensitive axis can be decreased greatly. On the other hand, the layout of the suspension beams also influences the thermal stress. The four anchors of the suspension beams should be arranged symmetrically, relative to the expansion center, and be close to each other. Improved structure design is illustrated schematically in Figure 2, where the condition *δ* = 0 holds.

**Figure 3:**
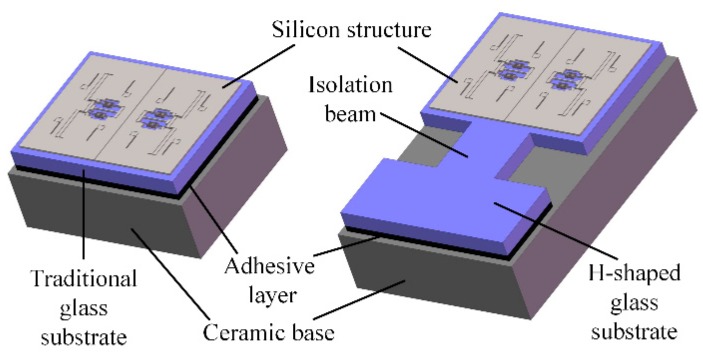
Comparison of micromachined resonant accelerometers (MRAs) with the traditional substrate and H-shaped isolation substrate.

In addition, a dedicated H-shaped isolation structure is proposed to reduce the thermal stress produced by the adhesive layer during the die attach process, as shown in Figure 3. Compared with a traditional rectangular glass substrate, the H-shaped glass substrate has a narrow isolation beam. One side of the H-shaped substrate is attached to the ceramic base via adhesive. The other side, which provides the bonding area of the silicon structure, is separated from the adhesive layer by the isolation beam. In this way, the silicon structure and the glass substrate below can virtually expand or shrink freely without the constraint of the ceramic base.

**Figure 4:**
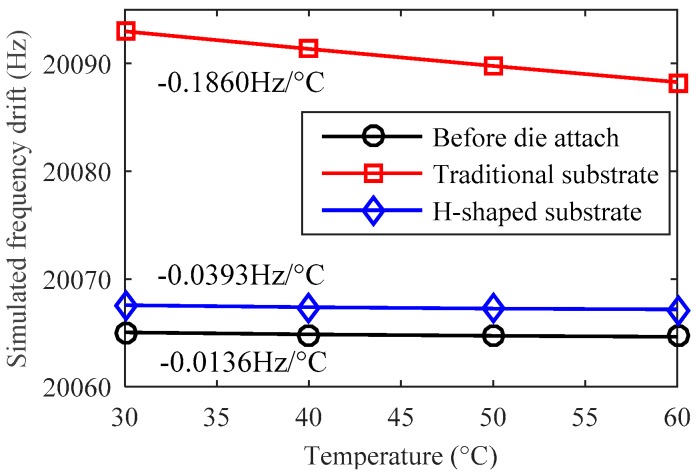
The simulated temperature drift of a single resonator’s natural frequency dominated by the thermal stress in design A.

**Figure 5:**
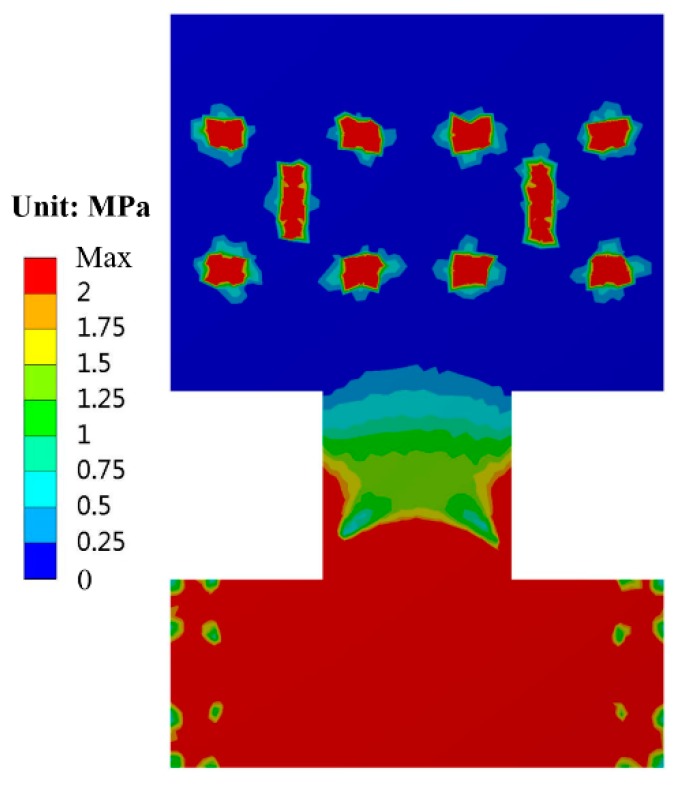
The simulated thermal stress distribution of the H-shaped substrate after die attach.

The temperature characteristics of design A are simulated by finite element analysis. To focus on the effect of the thermal stress, the Young’s modulus of all the materials are set to be constant while the temperature changes. The temperature drifts of the resonant frequency are respectively simulated before and after the glass substrate is fixed on the ceramic base by an adhesive. The simulation results show that the theoretical temperature drift caused by the thermal expansion of silicon and glass is only −0.0136 Hz/°C before die attach, as shown in Figure 4. After die attach, the frequency drift will increase up to −0.1860 Hz/°C if we use a traditional rectangular glass substrate. However, the frequency drift coefficient will change a little bit more, only −0.0393 Hz/°C if the H-shaped substrate is applied. The simulated thermal stress distribution of design A is shown in Figure 5. The finite element simulation results indicate that the H-shaped substrate can effectively isolate the thermal stress generated by the adhesive layer and the ceramic base. The thermal stress attenuates rapidly in the narrow isolation beam so that the residual stress in the silicon structure area can maintain a low level.

## 4. Design B: Single Anchored Isolation Frame

Although optimizing the location of anchors can remove most of the thermal stress along the sensitive *x*-axis, the stress along the *y*-axis still exists. Moreover, the H-shaped glass substrate can effectively decrease the package stress at the expense of extra processing steps, such as laser cutting and chip cleaning, which will also cause yield loss.

**Figure 6:**
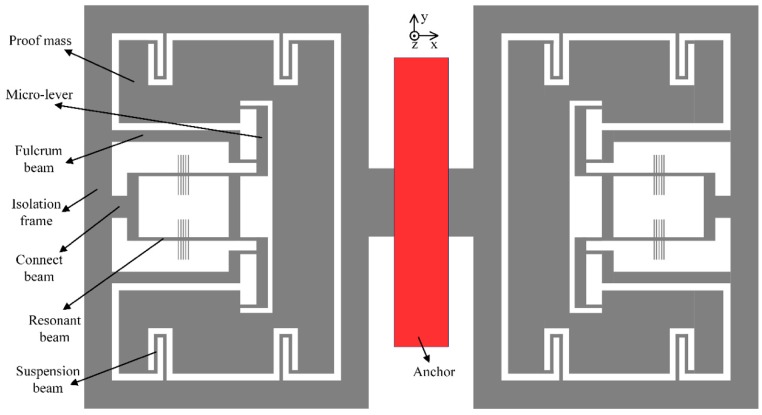
The schematic structure with single-anchored isolation frame.

Alternatively, a novel structure is further proposed to reduce the thermal stress and avoid the disadvantages of design A. In design B, all the traditional anchors of the suspension beams and micro-levers are removed and replaced by a rectangular isolation frame, as illustrated in Figure 6. Note that the structure is symmetric and the whole movable silicon structure is bonded to the substrate through a single anchor located in the center. It is noted that the suspension beams are directly connected to the isolation frame. The fulcrum of the micro-lever is fixed to the frame by a relatively wide and long beam. The inverted beam is no longer necessary and the resonator is connected to the other side of the frame away from the anchor.

**Figure 7:**
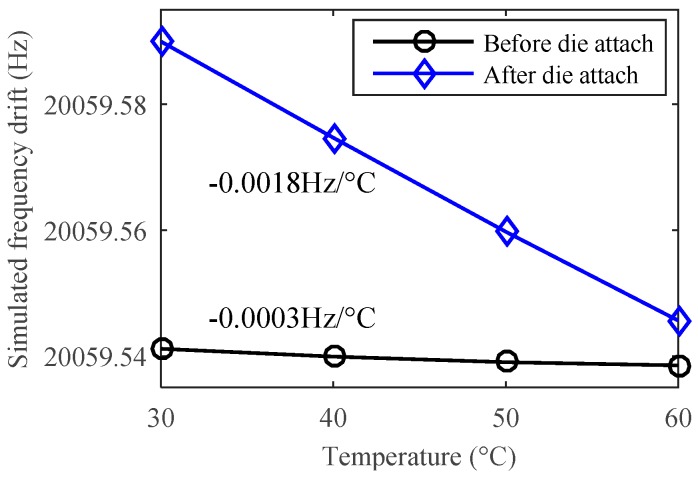
The simulated temperature drift of a single resonator’s natural frequency dominated by the thermal stress in design B.

**Figure 8:**
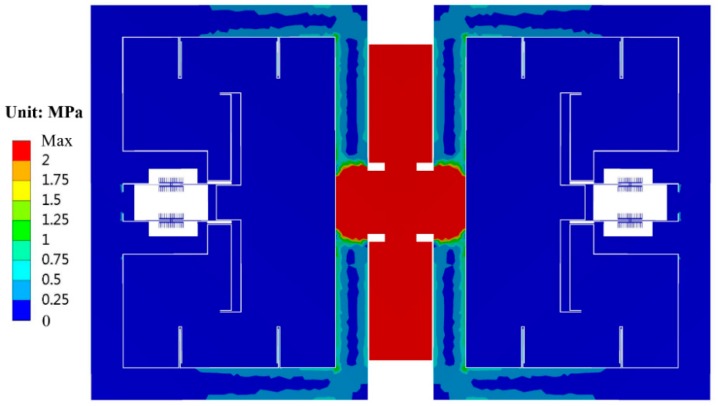
The simulated thermal stress distribution of the silicon structure after die attach in design B.

Since the single anchor will not generate relative location change as above multiple anchors may occur, in principle the structure in design B can attenuate the thermal stress both from the substrate and the die attach process. Finite element simulation shows that the theoretical temperature drift caused by the thermal expansion of silicon and glass is only −0.0003 Hz/°C, as shown in Figure 7. This result is much smaller than that in design A (−0.0136 Hz/°C). After die attach, the frequency drift will be only −0.0018 Hz/°C, even using a traditional rectangular substrate. The simulated thermal stress distribution of design B is shown in Figure 8. Considering the desirable bonding strength, the anchor area should not be too small, e.g., 1.72 mm^2^ in this work. The thermal stress is mainly generated inside the anchor area. Since the sensitive resonator and the suspension beams are configured to be far away from the anchor, the thermal stress in the resonant beam is negligible.

In designs A and B, the frequency drift caused by the Young’s modulus itself is simulated to be −0.5598 Hz/°C. This will dominate the total temperature drift of each single resonator. Benefiting from the full symmetric design of two resonators, the temperature drift of the differential frequency output will be reduced greatly.

Besides, the simulated modal frequencies of the H-shaped substrate and the single anchor frame are higher than 7.2 kHz and 1.7 kHz respectively, which are much larger than the operating bandwidth of the MRA (typically less than 0.5 kHz). Therefore, the MRA measurement is virtually undisturbed by these vibration modes of the two isolation structures.

## 5. Fabrication and Experiments

**Figure 9:**
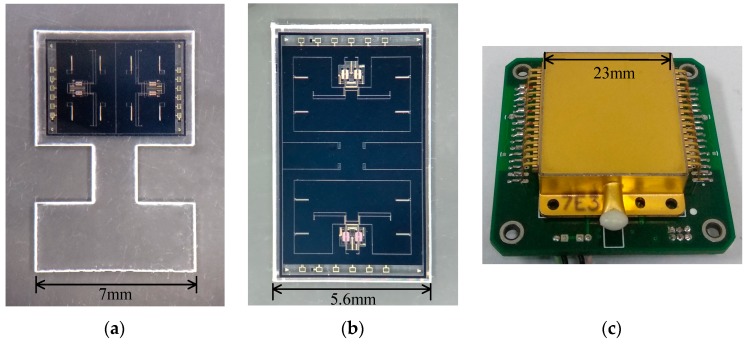
Photographs of the fabricated devices: (**a**) the structure in design A, (**b**) the structure in design B, (**c**) the vacuum packaged MRA device on the interface circuit board.

The MRA devices designed by both methods A and B were fabricated and tested experimentally. The silicon-glass structure was sealed in a hermetic metallic package and maintained at a high vacuum by a getter inside the package. The photographs of the SOG devices and associated package are shown in Figure 9. The degree of vacuum maintained inside the package is estimated at 0.1 Pa and the measured Q-factor of the resonator is up to 2.5 × 10^5^. The device is wire-bonded through the bond pads distributed on the edges of the glass substrate.

**Figure 10:**
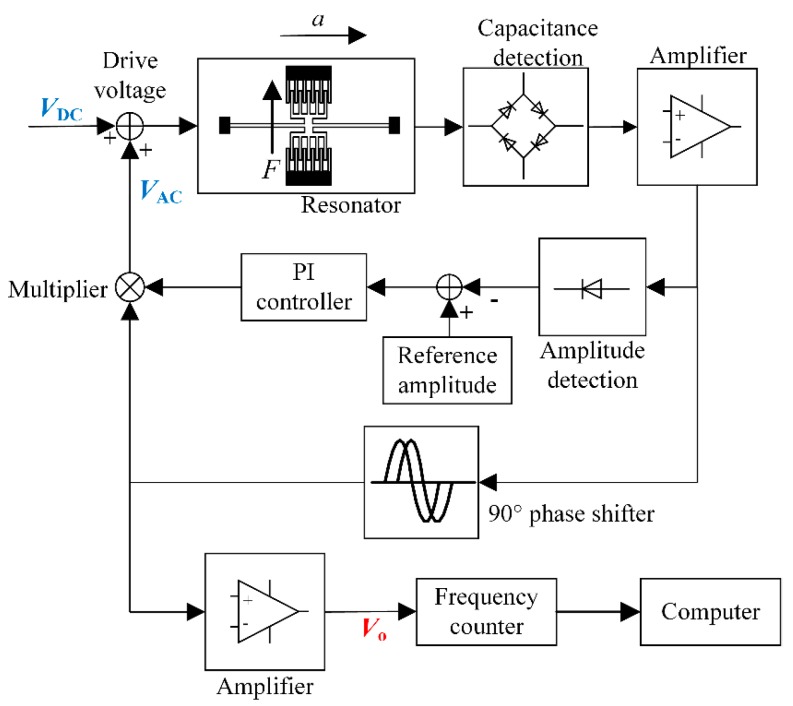
The block diagram of the MRA interface electronics.

The block diagram of the interface electronics is shown in Figure 10. The vibration of the resonator will cause the change of the capacitance between the comb teeth. Then the differential capacitance is detected by a ring-type diode based demodulation circuit [30]. The vibration amplitude is stabilized by automatic amplitude control (AAC). With a 90° phase shifter, the self-oscillation loop is established so the resonator will be locked at its resonant frequency. The frequency signal is captured by a commercial frequency counter (53230A, Keysight Technologies, Santa Rosa, CA, USA) and recorded by a computer in the following tests.

**Figure 11:**
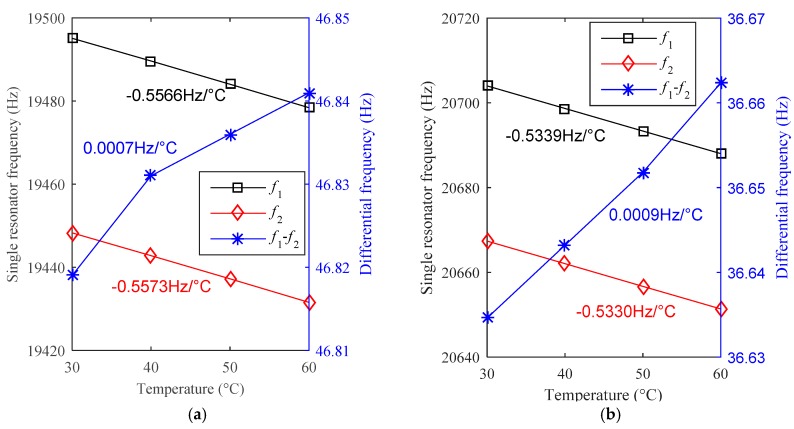
The measured temperature drifts of two MRA prototypes: (**a**) prototype A1 designed by method A, (**b**) prototype B1 designed by method B.

The measured temperature drift of three types of MRA devices (A1~A2, A3~A4 and B1~B2) are shown in Table 1. The devices A1~A4 are designed by method A and the devices B1~B2 are designed by method B. Note that the scale factors (SFs) of these devices vary widely because the force amplification factors of the micro-lever system are different in design. Imperfect fabrication contributes to the SF discrepancy in the same design of devices. Considering the full scale range of these accelerometers are designed to be greater than ±15 g, the scale factors listed in Table 1 were measured by setting the input axis of the accelerometer to different gravity vectors of +1 g and −1 g, respectively. In addition, *f*_1_ and *f*_2_ are the resonant frequency of the two resonators in one MRA device, respectively. Let TCF1 and TCF2 be the temperature coefficients of the frequency of the two resonators, the differential temperature coefficient of acceleration output is calculated by TCA = (TCF1 − TCF2)/SF. The measured TCF of a single resonator ranges from −0.5924 Hz/°C to −0.5330 Hz/°C, which agrees well with the simulated predication (−0.5598 Hz/°C). The sizes of all the resonators were designed to be the same while imperfect fabrication in different batches could cause the discrepancy. Considering the SF results, all the differential TCAs are lower than 13.9 μg/°C and the best TCA reaches as low as 1.9 μg/°C. The temperature drifts of the prototypes A1 and B1 are shown in Figure 11. The experimental results confirm the two proposed structures are effective to reduce thermal stress. The residual temperature drift is mainly caused by the fabrication discrepancy of the two resonators.

**Figure 12:**
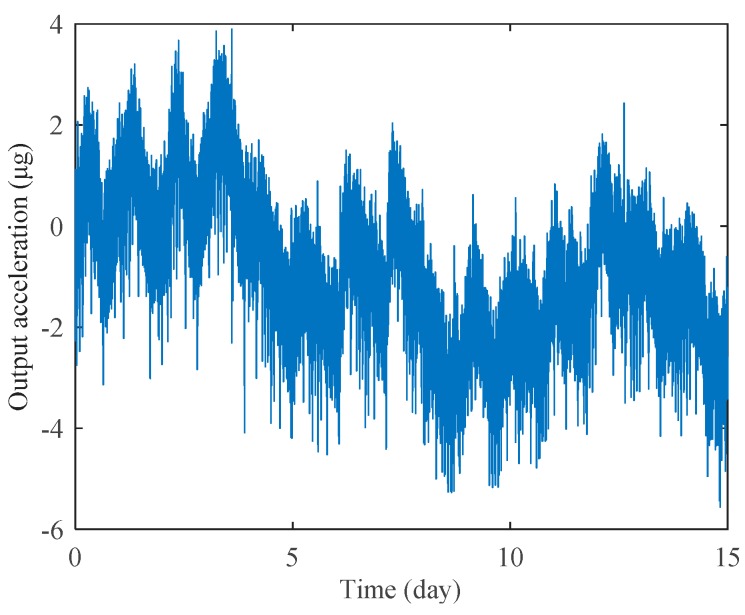
Long-time bias stability measurement of prototype A1 under room temperature.

Long-term bias stability is one of the most crucial performance requirements for high-precision accelerometers. We have recorded the bias stability of the prototype A1 for 15 days, as shown in Figure 12. The device is mounted at 0 g position and the test environment is under room temperature. The frequency output is acquired at a sampling rate of 1 Hz, where no temperature compensation or data smoothing is used in the plotted curves. After the MRA reached thermal equilibrium within two hours, the continuous measurement lasted 15 days. The standard deviation of the 15-day data is 1.4 μg, which is the lowest bias stability ever reported for MEMS accelerometers without temperature compensation or temperature control. The measured results indicate that the MRAs presented in this work exhibit expected temperature-insensitive characteristics and excellent long-term stability.

**Figure 13:**
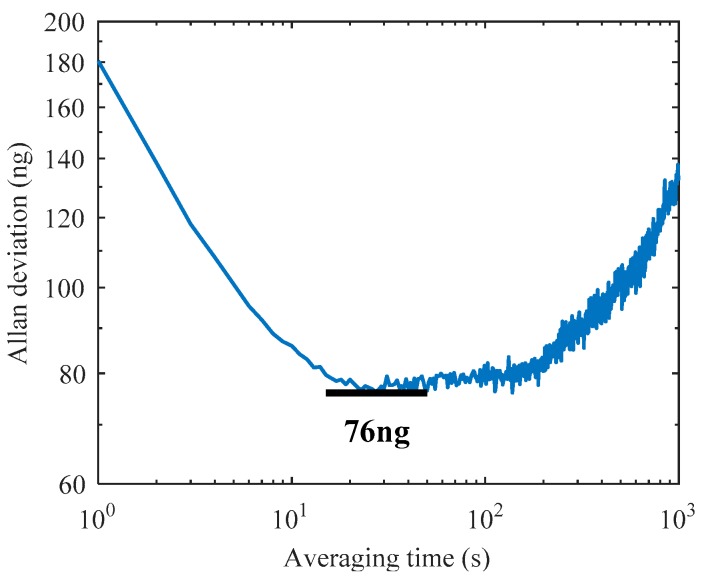
Allan deviation measurement of prototype A1.

The Allan deviation is a generally used method to evaluate the noise and drift error of various accelerometers. The minimum of the Allan deviation indicates the bias instability which limits the attainable resolution of the device. Along with the low noise design [31], the MRA shows high overall performance in Allan deviation measurement, as shown in Figure 13. The sampling frequency is set at 1 Hz and the total number of recorded data points is 60,000. The result shows a bias instability of 76 ng at 27 s averaging time, which is the best bias instability among reported MEMS resonant accelerometers.

## 6. Conclusions and Discussion

This paper presents two MRA structure designs to reduce the thermal stress effect. One design optimizes the anchor locations and utilizes an H-shaped isolation glass substrate instead of a classical rectangular substrate. The second design utilizes a single-anchored isolation frame to replace all the anchors connected to the suspension beams and micro-levers. Experimental results show that both of the two methods can effectively reduce the thermal stress generated in the microfabrication and device packaging process. The temperature drift coefficient of acceleration measurements is reduced down to 1.9 μg/°C. The bias stability is greatly improved where the 15-day result is 1.4 μg and the bias instability reaches 76 ng. The proposed structures offer a promising solution for the design of temperature-insensitive MRA devices. The experimental results are attractive for high-precision navigation and seismic measurement applications. In order to evaluate overall performance potential, future work will attempt to measure the Earth tides using our MRA prototypes.

## Figures and Tables

**Table 1:** Temperature drift of three types of MRA devices.

Serial Number	*f*_1_ (Hz)	*f*_2_ (Hz)	TCF1 (Hz/°C)	TCF2 (Hz/°C)	SF (Hz/g)	TCA (μg/°C)
A1	19,495	19,450	−0.5566	−0.5573	364	−1.9
A2	19,516	19,524	−0.5563	−0.5547	361	−4.4
A3	20,527	20,517	−0.5907	−0.5873	244	−13.9
A4	20,528	20,584	−0.5897	−0.5924	256	10.5
B1	20,704	20,667	−0.5339	−0.5330	203	4.4
B2	21,008	20,976	−0.5369	−0.5355	196	7.1
